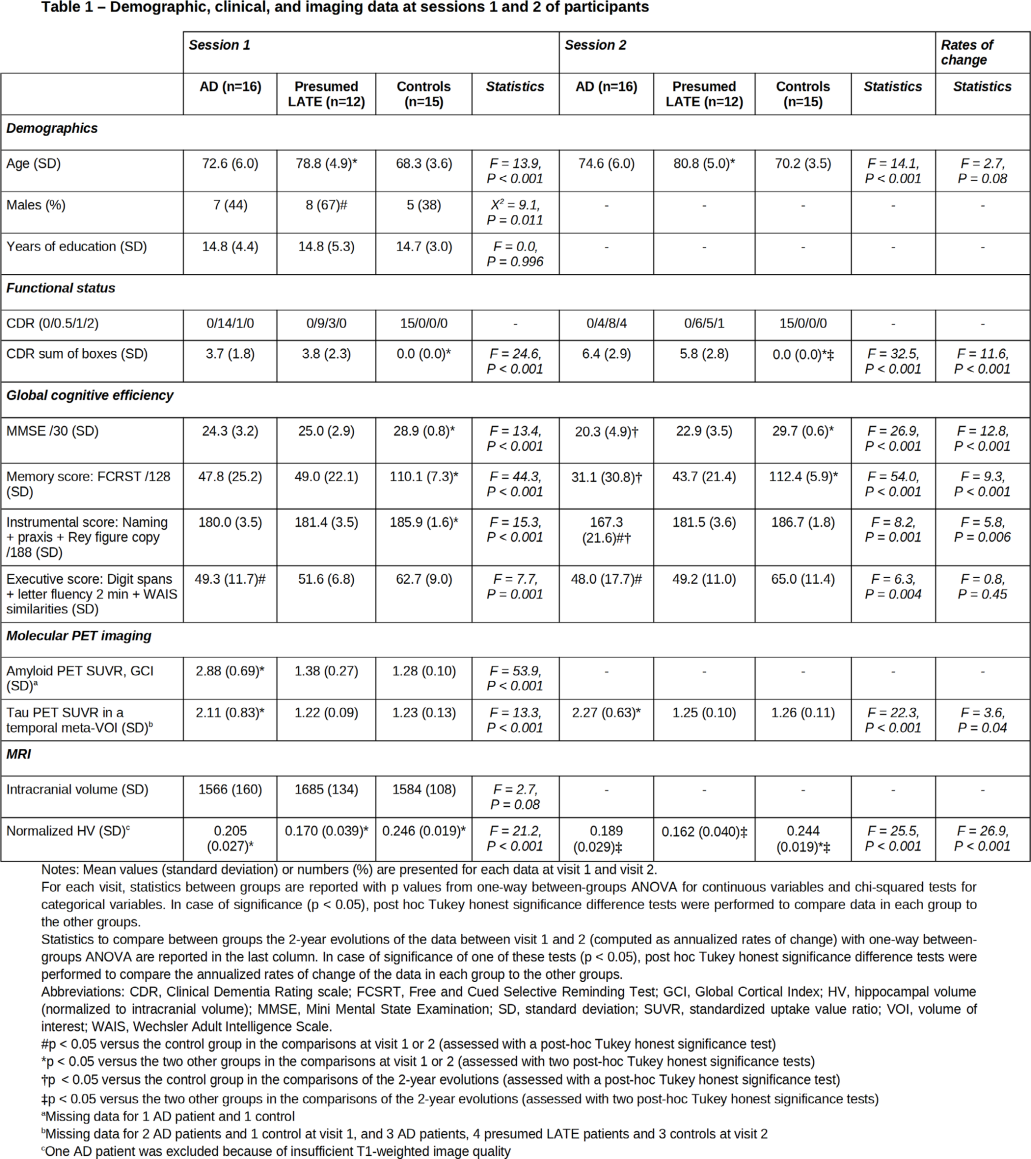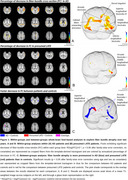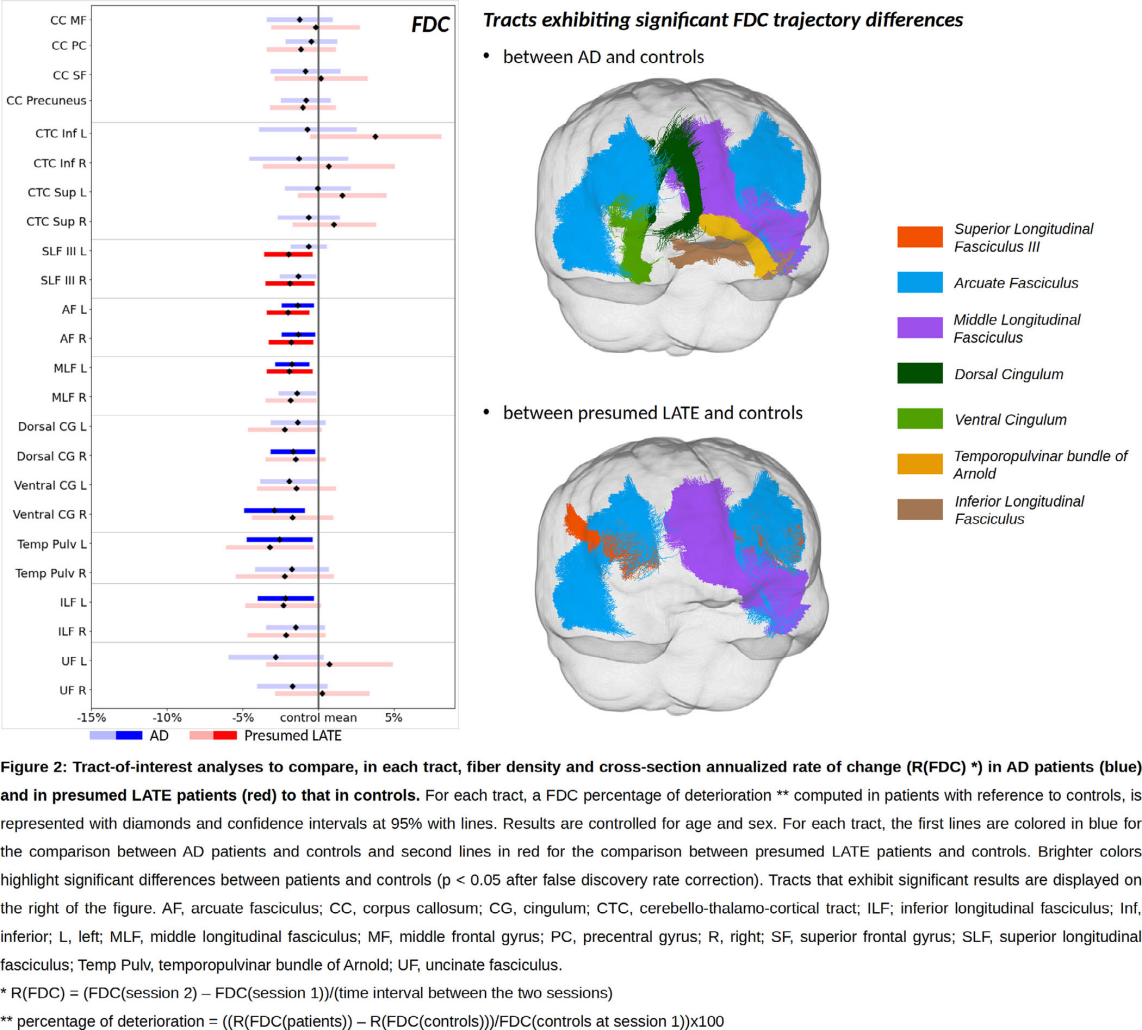# Longitudinal investigation of fiber bundle alteration in amnestic Alzheimer's disease and presumed LATE: a fixel‐based analysis

**DOI:** 10.1002/alz70856_102665

**Published:** 2025-12-24

**Authors:** Aurélie Lebrun, Yann Leprince, Julien Lagarde, Pauline Olivieri, Michel Bottlaender, Marie Sarazin

**Affiliations:** ^1^ Université Paris‐Saclay, CEA, NeuroSpin, UNIACT, Gif‐sur‐Yvette, France; ^2^ Université Paris‐Saclay, CEA, CNRS, Inserm, SHFJ, BioMaps, Orsay, France; ^3^ Université Paris Cité, Paris, France; ^4^ Neurology of Memory and Language Department, GHU Paris Psychiatrie & Neurosciences, Hôpital Sainte‐Anne, Paris, France

## Abstract

**Background:**

Typical Alzheimer's disease (AD) and Limbic‐predominant Age‐related TDP‐43 Encephalopathy (LATE) are neurodegenerative diseases that present with a similar amnestic syndrome but different proteinopathies. While these diseases are usually described as cortical diseases, our previous cross‐sectional study reported white matter (WM) fiber bundle alterations in both diseases with distinct patterns, suggesting a possible role of WM damage in these diseases. To further test this hypothesis, in this work we investigated the evolution of these alterations after two years in early AD and presumed LATE using high‐quality diffusion MRI and fixel‐based analysis.

**Method:**

We included 16 AD patients, 12 presumed LATE patients, and 15 healthy controls based on strict clinical and pathophysiological criteria (CSF biomarkers and amyloid and tau PET imaging) (Table 1). All participants underwent two 3‐tesla brain MRIs two years apart. Using multi‐shell diffusion MRI, we first performed whole‐brain fixel‐based analyses to investigate the progression of fixel metrics over two years within each group of patients (no covariates) and between patients and controls (covariates: age and sex). We then performed tract‐based analyses, using age and sex as covariates, to compare the rate of change in fiber density and cross‐section (FDC) between patients and controls at the level of tracts of interest that were previously identified in the whole‐brain analyses and in our previous cross‐sectional study.

**Result:**

We found that damage to WM fiber bundle progressed in both patient groups after two years (Figure 1), and that this decline was more marked in patients than in controls in tracts connecting the temporal lobe to the parietal and frontal lobes (arcuate fasciculus and middle longitudinal fasciculus) (Figure 2). In addition, AD patients exhibited a more pronounced decline than controls in the cingulum, the inferior longitudinal fasciculus and the temporopulvinar bundle of Arnold, while presumed LATE exhibited a more pronounced decline in the ventral section of the superior longitudinal fasciculus.

**Conclusion:**

These results show that WM fiber bundle alterations are not only detectable in patients with AD and presumed LATE but that they worsen over time, more markedly than in controls, underscoring the importance of WM fiber bundle alterations in these diseases.